# Metformin enhances protection in guinea pigs chronically infected with *Mycobacterium tuberculosis*

**DOI:** 10.1038/s41598-020-73212-y

**Published:** 2020-10-01

**Authors:** Jessica D. Haugen Frenkel, David F. Ackart, Alexandra K. Todd, James E. DiLisio, Siana Hoffman, Samantha Tanner, Dilara Kiran, Megan Murray, Adam Chicco, Andrés Obregón-Henao, Brendan K. Podell, Randall J. Basaraba

**Affiliations:** 1 Department of Microbiology, Immunology and Pathology, Metabolism of Infectious Diseases Laboratory, Mycobacteria Research Laboratories, College of Veterinary Medicine and Biomedical Sciences, Colorado State University, Fort Collins, CO USA; 2 Department of Global Health and Social Medicine, Harvard Medical School, Boston, MA USA; 3 Department of Biomedical Sciences, College of Veterinary Medicine and Biomedical Sciences, Colorado State University, Fort Collins, CO USA

**Keywords:** Health sciences, Pathogens

## Abstract

Tuberculosis (TB) is a chronic inflammatory disease that is often associated with alterations in systemic and cellular metabolism that resolves following successful antimicrobial drug treatment. We hypothesized that altered systemic glucose metabolism as a consequence of *Mycobacterium tuberculosis* (Mtb) infection, contributes to TB pathogenesis, and when normalized with anti-glycemic drugs would improve clinical outcomes. To test this hypothesis, guinea pigs were treated daily with the anti-diabetic drug metformin starting 4 weeks prior or concurrent with aerosol exposure to the H37Rv strain of Mtb. In the chronic stages of infection, Mtb infected metformin-treated animals had restored systemic insulin sensitivity but remained glucose intolerant as determined by oral glucose tolerance testing. Despite persistent glucose intolerance, metformin-treated guinea pigs had a 2.8-fold reduction in lung lesion burden and a 0.7 log decrease in CFUs. An alternative hypothesis that metformin treatment improved clinical disease by having a direct effect on immune cell energy metabolism was tested using extracellular flux analysis and flow cytometry. The proinflammatory immune response to Mtb infection in untreated guinea pigs was associated with a marked increase in energy metabolism (glycolysis and mitochondrial respiration) of peripheral blood mononuclear cells (PBMCs), which was normalized in metformin-treated guinea pigs. Moreover, both CD4^+^ and CD8^+^ T lymphocytes from Mtb infected, metformin treated animals maintained a more normal mitochondrial membrane potential while those isolated from untreated animals had persistent mitochondrial hyperpolarization. These data suggest that metformin promotes natural host resistance to Mtb infection by maintaining immune cell metabolic homeostasis and function during the chronic stages of active TB disease.

## Introduction

The persistent proinflammatory immune response associated with active *Mycobacterium tuberculosis* (Mtb) infection in some TB patients contributes to alterations in systemic glucose and lipid metabolism^1–3^. The role elevated blood glucose levels has on TB disease severity and progression has been best characterized in diabetic patients, which have a 1.5 to 3-fold increased risk for developing active TB disease and are twice as likely to die or develop reactivation of a latent Mtb infection^4–8^. However, the impact non-diabetic hyperglycemia has on TB pathogenesis is less well understood. To determine the impact non-diabetic hyperglycemia has on the pathogenesis of TB we treated guinea pigs with the anti-diabetic drug metformin prior to or concurrent with aerosol exposure to Mtb. Metformin is among the safest and most widely prescribed drugs used to control blood glucose levels in patients with type 2 diabetes mellitus (T2DM). Metformin helps lower and maintain more normal blood glucose levels in diabetic patients by improving systemic insulin sensitivity and lowering glucose production by the liver^9,10^. In the context of TB, several studies have suggested that Mtb infected individuals with T2DM taking metformin, have slower TB disease progression and improved clinical outcomes and survival compared to individuals not treated with metformin or with poor diabetes control^11–13^.

In addition to its anti-glycemic effects, metformin has also been widely studied as an immunomodulatory drug for the treatment of various other communicable and non-communicable diseases^14^, with observed clinical improvement in patients with cancer, a variety of chronic bacterial and viral infections as well as autoimmune diseases such as systemic lupus erythematosus (SLE)^15–20^. Although the exact mechanisms of action have not been determined, metformin has been shown to have a direct effect on immune cell metabolism, which is linked to T lymphocyte differentiation, function, and survival^21–26^. This suggests that in addition to normalizing systemic glucose metabolic derangements caused by chronic inflammation, metformin may also modulate the function of immune cells that are important in the protection and pathogenesis of TB. We show here that metformin treatment of Mtb infected guinea pigs improved clinical disease in part by partially restoring systemic glucose metabolism as well as having a direct effect on cellular metabolism of immune cells that are important in the protection against TB.

## Results

### Metformin restores insulin sensitivity but not glucose intolerance during Mtb infection

**Figure 1:**
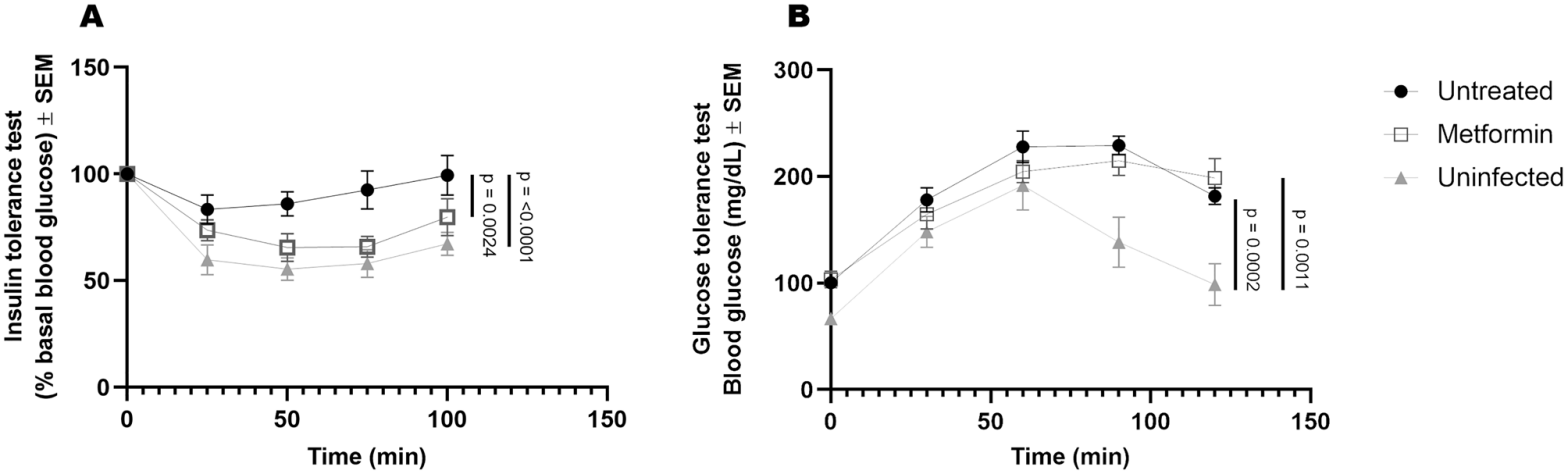
Metformin restores insulin sensitivity but not glucose tolerance during Mtb infection. Duncan-Hartley guinea pigs received oral administration of 25 mg/kg metformin or mock treatment once daily beginning 28 days prior to low-dose aerosol infection with Mtb H37Rv. Thirty days following infection guinea pigs were subjected to an insulin tolerance test by injecting 0.5 units/kg human recombinant insulin SC (**A**) or an oral glucose tolerance test by challenging fasted animals with a 2 g/kg bolus of d-glucose (**B**). Blood glucose concentrations were measured at 0, 25, 50, 75, and 100 min post insulin injection and 0, 30, 60, 90, and 120 min post oral glucose challenge. A 2-factor mix model was used to determine significances with Tukey’s correction for multiple comparisons. n = 9, ***p ≤ 0.001 ; **p ≤ 0.01.

Previously, human and animal studies have demonstrated that glucose intolerance, consistent with systemic insulin resistance, develops in response to chronic Mtb infection^1,3,27^. We hypothesized that insulin resistance is responsible for altered systemic glucose metabolism that is characteristic of chronic infection. We tested whether treatment with metformin initiated prior to or concurrent with Mtb infection restored systemic glucose metabolism. Guinea pigs infected with Mtb developed non-diabetic hyperglycemia (Fig. 1B, untreated vs. uninfected *p* = 0.0002) and systemic insulin resistance as determined by glucose and insulin tolerance testing, respectively (Fig. 1A, untreated vs. uninfected *p* < 0.0001). In contrast, guinea pigs treated with metformin maintained systemic insulin sensitivity comparable to uninfected animals (Fig. 1A and Supplemental Figure 1).

Consistent with previous findings in humans, oral glucose tolerance testing showed Mtb infected guinea pigs developed systemic glucose intolerance that was not reversed by metformin treatment (Fig. 1B and Supplemental Figure 2).

**Figure 2:**
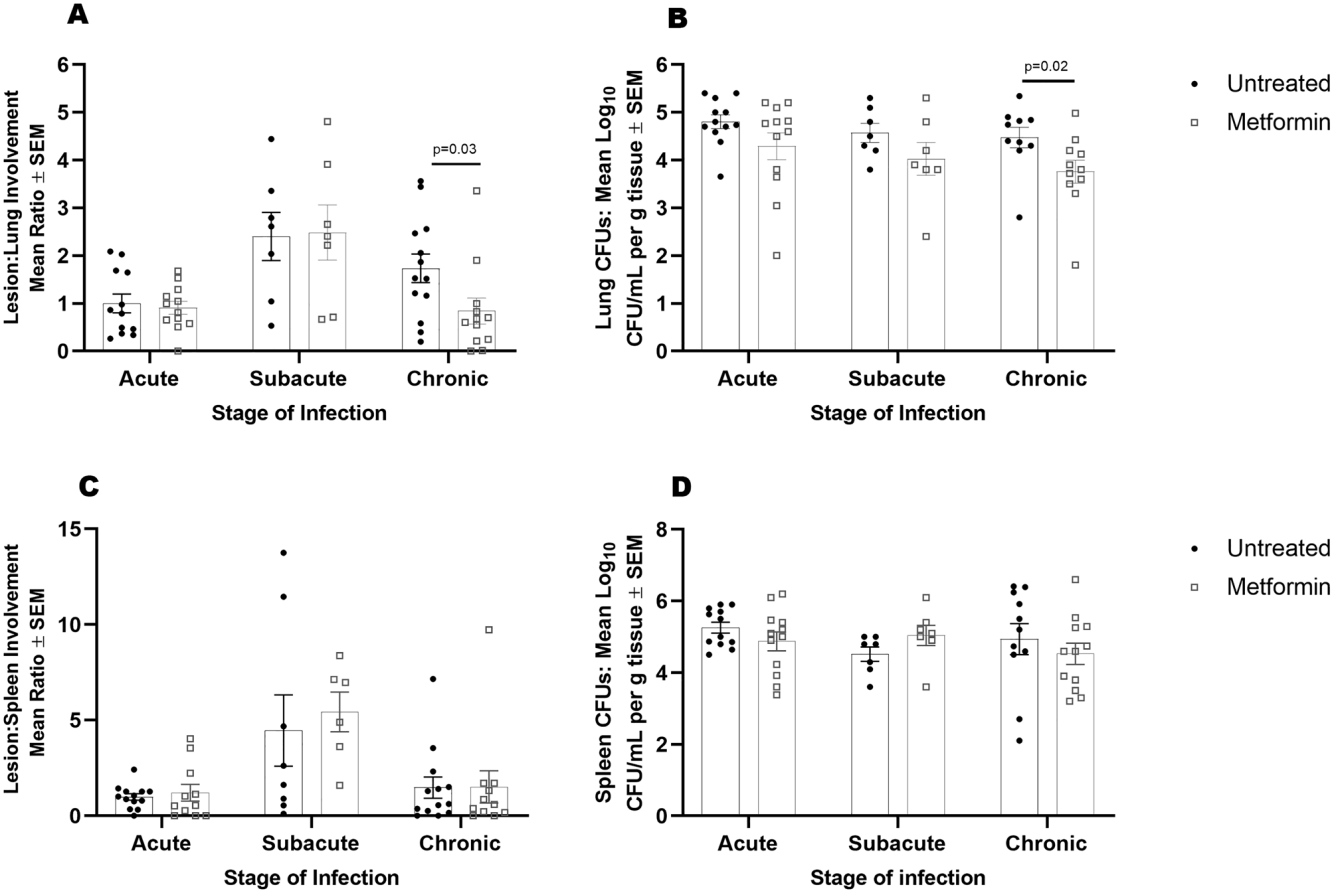
Metformin enhances host resistance to Mtb. During acute, subacute, and chronic stages of infection lungs and spleens were harvested from euthanized, Mtb infected untreated- and metformin-treated guinea pigs to evaluate lesion burden (**A**, **C**) and bacterial burden (CFUs) (**B**, **D**). Stereo Investigator software was used to calculate the percent of lesion compared to total section area in guinea pig lungs and spleens (**A**, **C**). Percent lesion involvement was normalized to acute untreated. Colony forming units were generated by plating homogenized (**C**) lung or (**D**) spleen tissue on 7H11 agar plates and were quantified per gram of tissue. n = 7–12 animals per group per timepoint. Two outliers were found using Tukey IQR method (**B**). All outliers were greater than 2.0 times the IQR. Mann–Whitney test was used to determine significances between treatments within stage of infection *p ≤ 0.05.

### Metformin enhances host resistance to Mtb

**Figure 3:**
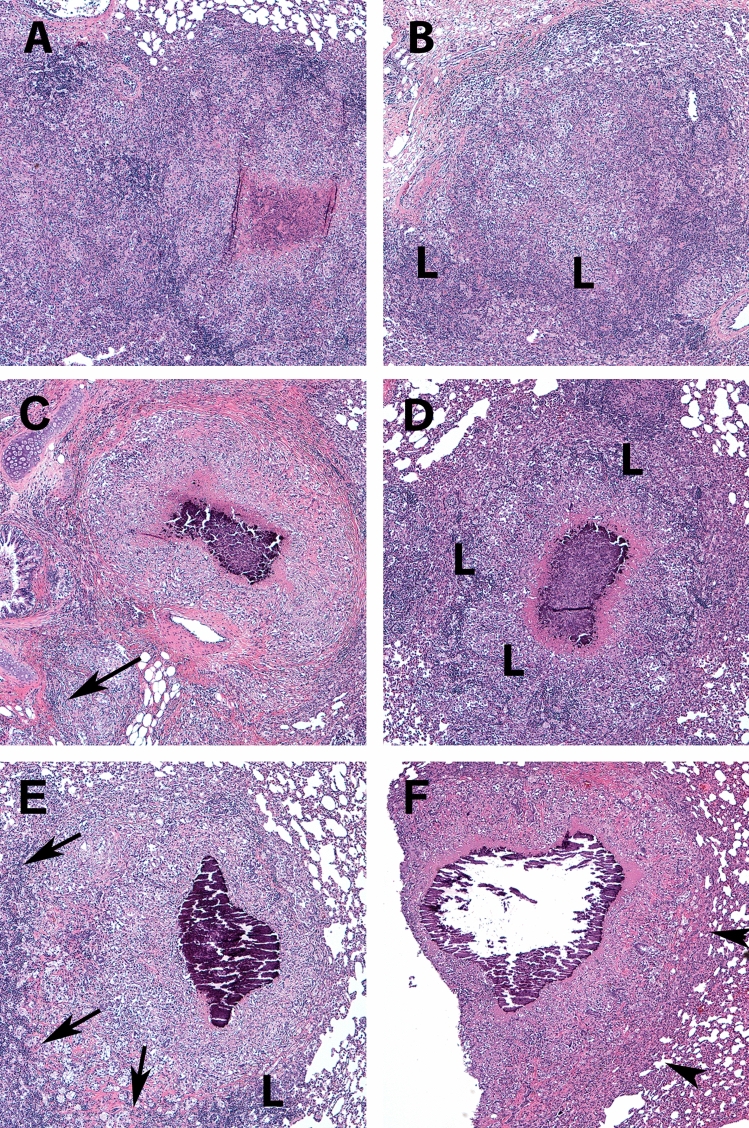
Metformin treatment of Mtb infected guinea pigs resolve lung lesions in the chronic stages of infection. Guinea pigs were treated orally with mock treatment (OraSweet) (**A**, **C**, **E**) or metformin (**B**, **D**, **F**) and euthanized during the acute (**A**, **B**), subacute (**C**, **D**) or chronic (**E**, **F**) stages of infection with the H37Rv strain of Mtb. The progression of disease from the acute to chronic stages of infection in mock treated animals showed progressive perilesional inflammation (arrows) that corresponded to a higher lung lesion burden. In contrast, in metformin treated guinea pigs, inflammatory cells in the acute and subacute stages of infection are predominately lymphocytes (L) with less peri-leional spread (arrowheads). In the chronic stages of infection in metformin treated animals the decreased overall lung lesion burden was accompanied by well-encapsulated granulomas that were sharply demarcated from the more normal lung parenchyma (arrowheads). (**H**) and (**E**) stain, 100× magnification.

To evaluate whether the effects of metformin on systemic metabolism influenced the outcome of TB disease severity, we quantified Mtb colony forming units (CFUs) and lesion burden at acute (21–30 days), subacute (45–60 days), and chronic (75–90 days) stages of infection. At acute and subacute timepoints, there were no differences in lesion burden nor CFUs in the lungs, spleens, or livers of infected animals (Fig. 2A–D, Supplemental Figure 3). During chronic stages of infection however, we observed a 2.8-fold decrease in lung lesion burden and 0.7 log10 reduction in CFUs in metformin-treated compared to untreated guinea pigs (Fig. 2A, *p* = 0.03 and B, *p* = 0.02). Lung tissue histology showed distinct differences in granuloma morphology in comparison to those from untreated animals. Granulomas from metformin-treated animals had a higher proportion of lymphocytes at acute and subacute time points that progressed to well-organized and delineated granulomas with less severe perilesional inflammation in the chronic stages of disease (Fig. 3A–E).

Clinical signs of disease were only modestly improved in metformin-treated animals with a less, but nonsignificant, increase in body temperature in metformin-treated compared to untreated animals (Supplemental Figure 3). There were no significant differences in body weights between treated and untreated animals over the course of infection (Supplemental Figure 3), and survival at 250 days was not significantly different between the treatment groups (Supplemental Figure 3).

### T cell metabolism is regulated by metformin

Given the effect of metformin in decreasing disease burden without fully restoring systemic glucose metabolism, we hypothesized that improved protection might be due to restoration of cellular rather than systemic metabolic alterations. To assess whether metformin modulated immune cell metabolism following Mtb infection, we evaluated metabolic profiles and the relative abundance of CD4^+^, CD8^+^, and MHCII^+^ cells among PBMCs from Mtb infected, treated and untreated guinea pigs. At all stages of infection, PBMCs were comprised of approximately 60% T cells with no significant changes in the proportions of phenotypes, regardless of treatment (Supplemental Figure 4). Metabolic profiles were assessed in PBMCs ex vivo using extracellular flux analysis, which measures the rate of oxidative phosphorylation (OXPHOS), expressed as oxygen consumption rate (OCR), and the rate of glycolysis, measured by the change in extracellular pH, expressed as extracellular acidification rate (ECAR).

**Figure 4:**
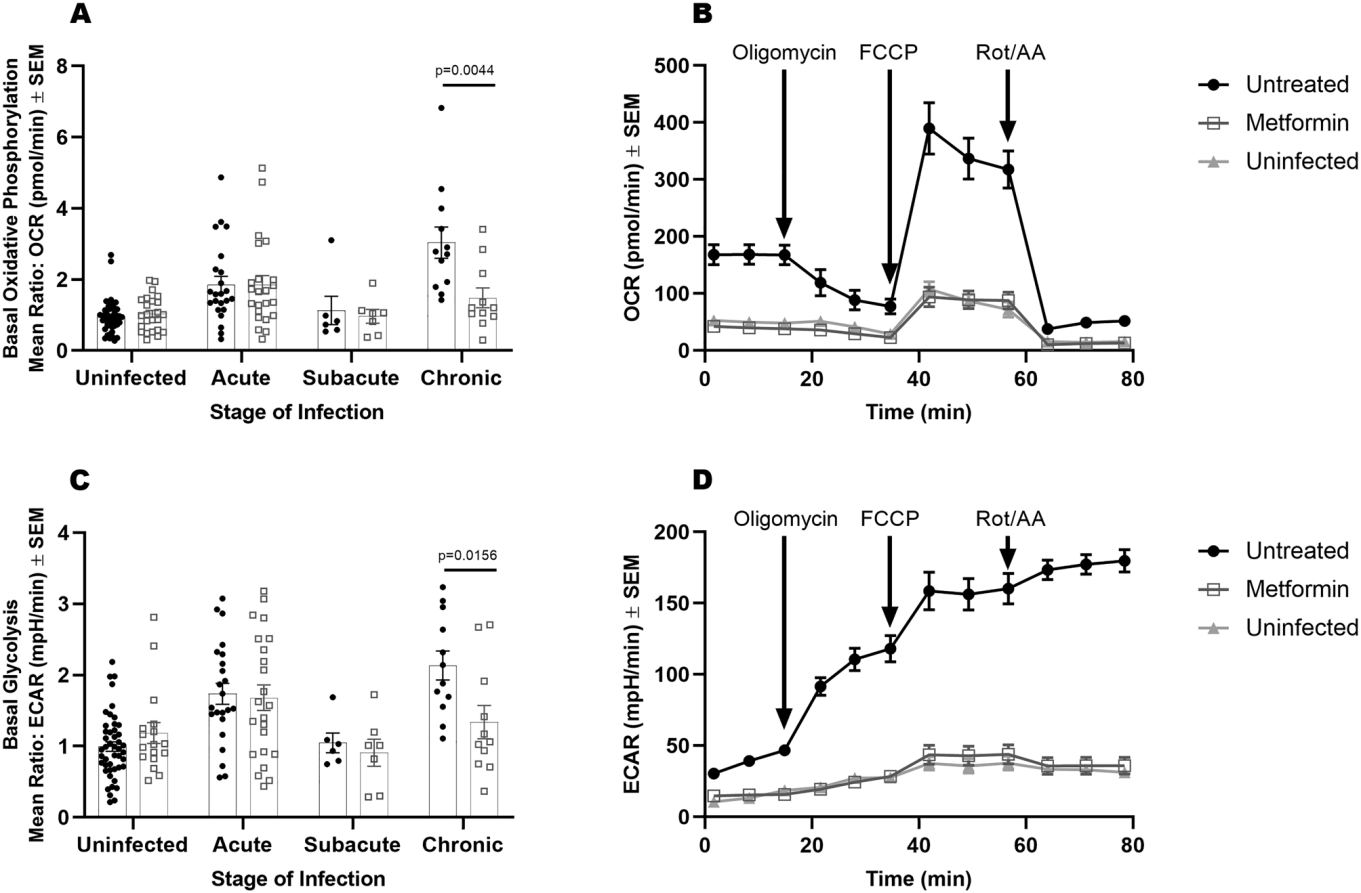
T cell metabolism is regulated by metformin. Live PBMCs were isolated from untreated or metformin-treated guinea pigs prior to or during acute, subacute, or chronic Mtb infection. Basal oxidative phosphorylation (OCR) (**A**) and basal glycolysis (ECAR) (**C**) were measured in live PBMCs by extracellular flux analysis. Data was normalized to the mean of the uninfected untreated group between two independent experiments. Mean values from a mitochondrial stress test performed 75 days post infection (**B**, **D**). Curves represent raw OCR (**B**) and ECAR (**D**) values generated in response to the addition of oligomycin, FCCP, or rotenone and antimycin A. Mann–Whitney test was used to determine significance between treatments within stage of infection. n = 24, *p ≤ 0.05 ; **p ≤ 0.01.

Following acute Mtb infection, dramatic increases in the utilization of OXPHOS and glycolysis were observed in both metformin-treated and untreated animals (Fig. 4A,C). During subacute infection, PBMC metabolic rates decreased to levels comparable to uninfected animals in both metformin treated and untreated groups (Fig. 4A,C). Following the onset of chronic infection however, PBMCs from untreated animals increased OXPHOS and glycolysis by roughly 3 and 2-fold, respectively. In contrast, PBMCs from metformin-treated guinea pigs maintained OXPHOS and glycolysis levels similar to that of uninfected guinea pigs during chronic infection (Fig. 4A–D, Basal OXPHOS *p* = 0.0044, Basal Glycolysis *p* = 0.0156).

### Metformin modulates T cell mitochondrial membrane potential and superoxide production

**Figure 5:**
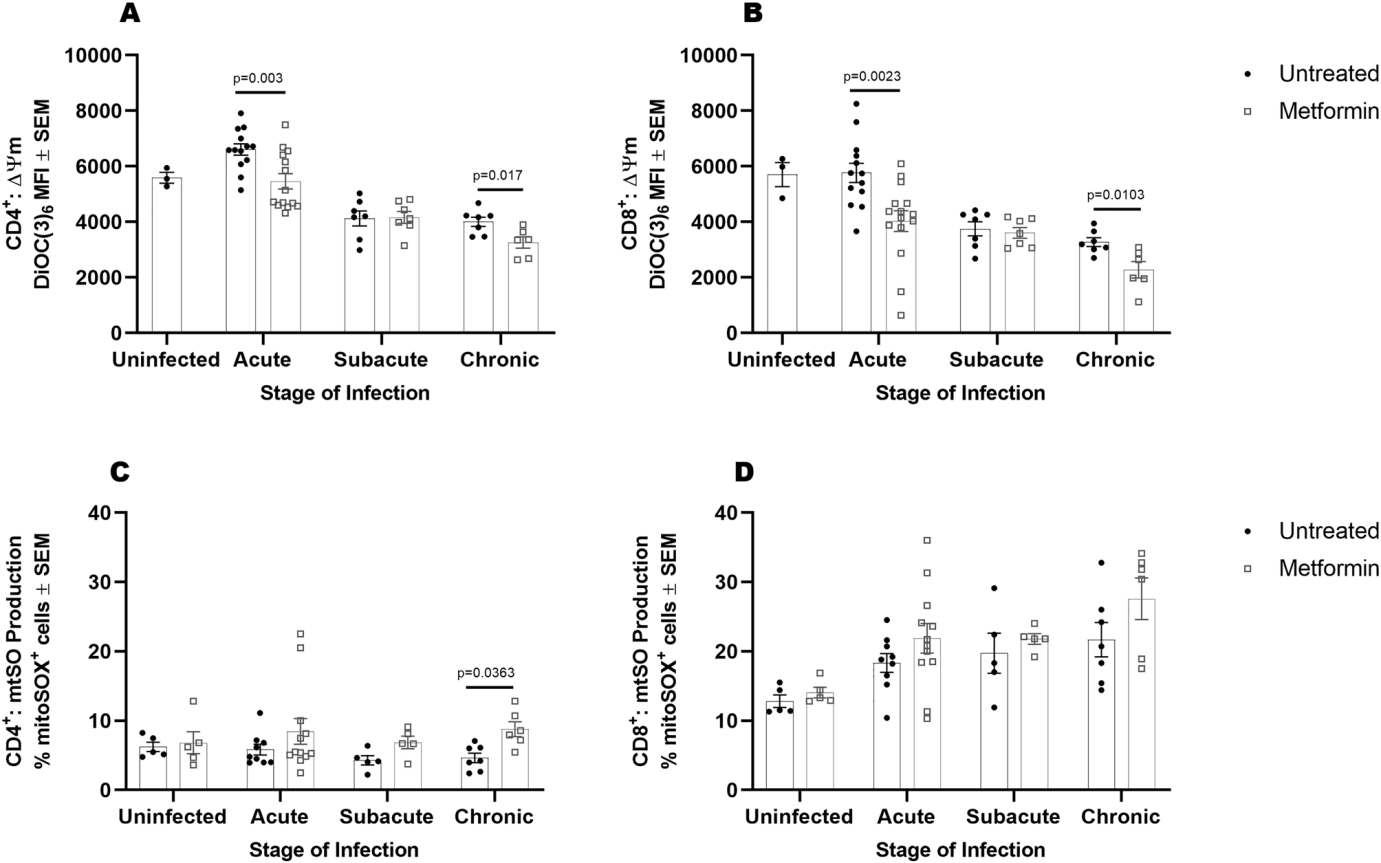
Metformin modulates T cell mitochondrial membrane potential and superoxide production. Peripheral mononuclear cells isolated from Mtb infected untreated and metformin-treated guinea pigs were stained with anti-CD4, anti-CD8, anti-MHC II, the ΔΨm dye DiOC(6)_3_ (25 nM), and the mitochondrial superoxide (mtSO) indicator dye MitoSOX prior to infection or during acute, subacute, and chronic stages of infection. Differential ΔΨm was determined between CD4^+^ (**A**) and CD8^+^ (**B**) by measuring the median fluorescent intensity of DiOC(6)_3_ by flow cytometry. Superoxide production was measured in CD4^+^ (**C**) and CD8^+^ (**D**) by mitoSOX staining and flow cytometry quantification. One outlier was identified using Tukey IQR method which was greater than 2.0 times the IQR (**A**). Unpaired t-test with Welch’s correction was used to determine significance between groups within stage of infection (**A**, **B**). A 2-factor ANOVA was used to determine significances with Sidak’s correction for multiple comparisons (**C**, **D**). n = 3–12 animals per group per timepoint, *p ≤ 0.05; **p ≤ 0.01.

Previous studies have shown that metformin reduces pathology associated with chronic inflammation in a mouse arthritis model by reprogramming metabolism, and thus function, of T lymphocytes^28,29^. In particular, mitochondrial membrane potential (ΔΨm) and mitochondrial superoxide (mtSO) are found to be heightened in persistently activated T cells in models of chronic inflammation, ultimately leading to cell death and oxidative damage^30,31^. To determine whether the differences in PBMC metabolic phenotypes are linked to alterations in T cell metabolism, we measured mitochondrial membrane potential and mitochondrial superoxide production in CD4^+^ and CD8^+^ T cells from PBMCs of Mtb-infected guinea pigs. During acute and chronic stages of infection, we observed significantly lower mitochondrial membrane potential in CD4^+^ and CD8^+^ T cells from metformin-treated guinea pigs compared to untreated animals (Fig. 5A, acute *p* = 0.003 and chronic *p* = 0.017; Fig. 5B, acute *p* = 0.0023 and chronic *p* = 0.0103). We ensured that the differences in membrane potential were not due to variations in mitochondrial size or numbers by measuring mitochondrial mass; and despite differences in membrane potential, mitochondrial mass remained consistent between both groups at all stages of infection (Supplemental Figure 5). We noted that mitochondrial superoxide was increased nearly twofold higher in CD4^+^ T cells from metformin-treated compared to untreated animals during chronic infection (Fig. 5C, *p* = 0.0363). Because superoxide is produced in response to oxidative stress, we measured the ratio of glutathione (GSH) to glutathione disulfide (GSSG) to determine whether an increase in superoxide resulted in response to cellular stress. Although superoxide was increased in CD4^+^ T cells from metformin treated animals during chronic infection, GSH: GSSG ratios were not different between groups (Supplemental Figure 5).

### Metformin decreases inflammatory cytokine production in the lung during Mtb infection

**Figure 6:**
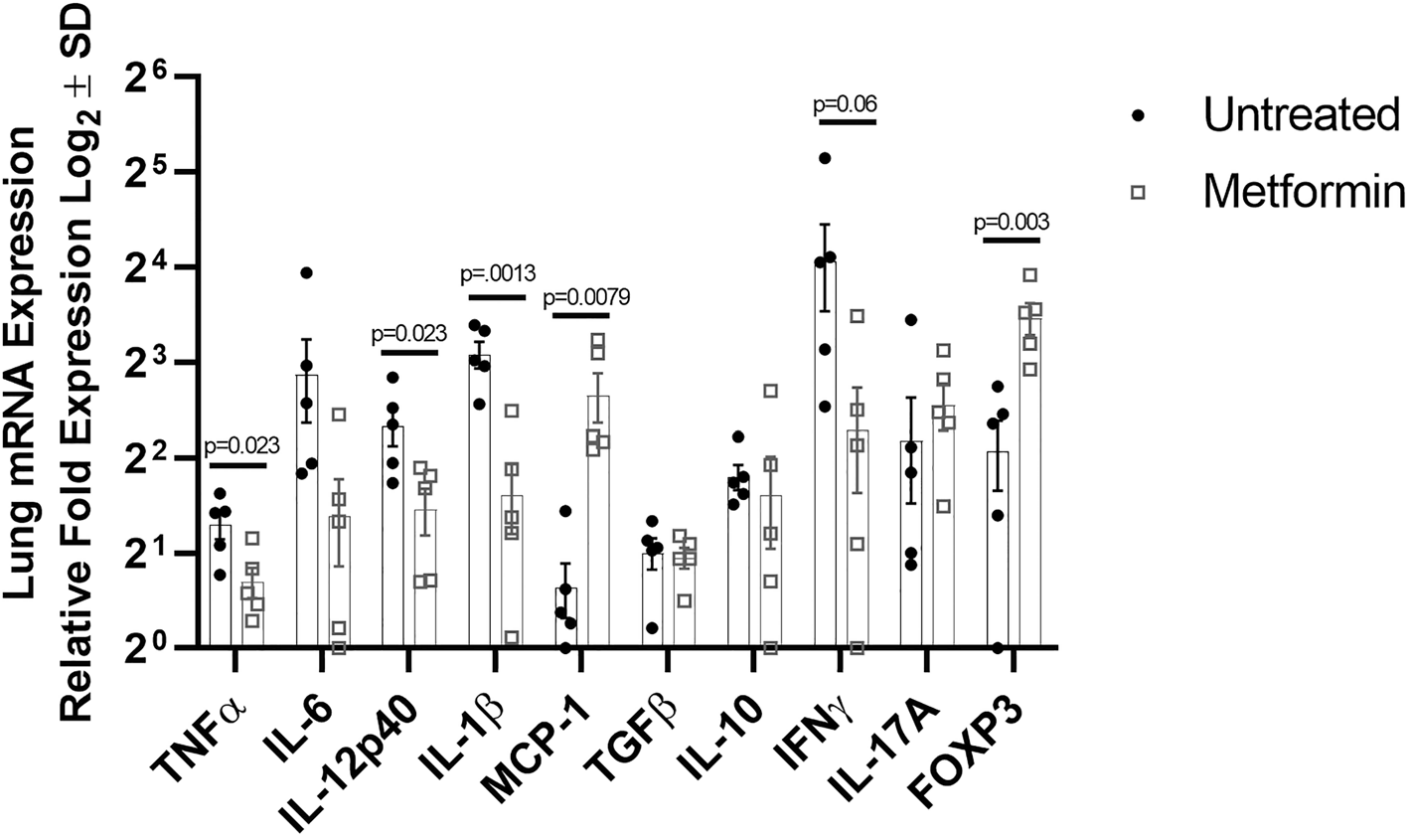
Metformin decreases inflammatory cytokine production in the lung during Mtb infection. RNeasy columns were used to extract RNA from homogenized lung tissue from chronically Mtb infected guinea pigs (day 75), which were either untreated or received metformin-treatment. 1 μg RNA was converted to cDNA and primers against TNFα, IL-6, IL-12p40, IL-1β MCP-1, TGFβ, IL-10, IFNγ, IL-17A, and FOXP3 were used to quantify gene expression by qRT-PCR. Unpaired Student’s t-test with Welch’s correction or Mann–Whitney test was used to determine significances with Bonferroni correction for multiple comparisons. n = 5, *p ≤ 0.05; **p ≤ 0.01.

To further understand the differences observed between disease burden and granuloma morphology in response to metformin treatment, we sought to evaluate cytokine and transcription factor gene expression in granulomas from untreated and metformin-treated animals during chronic Mtb infection. In comparison to metformin-treated animals, untreated guinea pigs displayed more production of the pro-inflammatory cytokines TNFα (*p* = 0.023), IL-12p40 (*p* = 0.023), and IL-1β (*p* = 0.0013), and enhanced production of IFN-γ and IL-6, though not statistically significant (Fig. 6). Furthermore, metformin-treated animals displayed significantly higher expression of the regulatory T cell transcription factor FOXP3 (*p* = − 0.003), however there were no significant differences in the abundance of anti-inflammatory IL-10 or TGF-β. Additionally, we observed a significant increase in monocyte chemoattractant protein (MCP-1, *p* = 0.0079) in granulomas from metformin compared to untreated animals.

## Methods

### Animals and sample collection

Four week old, female, outbred Dunkin–Hartley guinea pigs, n = 14 per group, were purchased from Charles River Laboratories and housed in a biosafety level 3 laboratory at the Colorado State University Laboratory Animal Resources facility accredited by the American Association for Accreditation of Laboratory Animal Care (AAALAC). All animal experiments were performed in accordance with the National Research Council's Guide for the Care and Use of Laboratory Animals and were approved by the Animal Care and Usage Committee at Colorado State University.

Animals were chipped (BioMedic Data Systems, Inc.) for animal identification and continuous recording of body temperature. For serial blood collection, guinea pigs were anesthetized via isoflurane inhalation and blood collected by percutaneous venipuncture of the cranial vena cava. At the time of euthanasia, guinea pigs were administered 50 mg/kg of ketamine and 5 mg/kg of Xylazine via intramuscular injection for anesthetic induction. Under terminal anesthesia, blood was collected, and guinea pigs were euthanized by IP overdose of pentobarbital. Tissues were collected for histopathology by fixing in 4% paraformaldehyde or stored at − 80 °C for homogenization and quantification of Mtb.

### Metformin treatment

Guinea pigs received oral administration of either 20 mg/kg of metformin (Spectrum Chemical MFG Corp, New Brunswick, NJ), purchased through the CSU Veterinary Teaching Hospital Pharmacy, in a carrier suspension of 50% ORA-sweet® (Perrigo) 50% water or mock treatment, carrier suspension of 50% ORA-sweet® (Perrigo) 50% water, daily beginning 28 days prior to or concurrent with Mtb infection. Dosage was chosen based on the human equivalent serum concentration of ~ 10 μM, confirmed by pharmacokinetic analysis (Figure S6). Treatment was continued once daily throughout the duration of the study.

### Mtb aerosol exposure

Culture stocks of Mtb strain H37Rv (TMC #102, Trudeau Institute) were agitated in glass test tubes, collected at an OD600 nm between 0.8 and 1.0, and frozen at − 80 °C in Proskauer-Beck liquid medium containing 0.05% Tween-80. Titer was determined and bacteria were diluted in water to 1 × 10^6^ CFU/ml. Approximately 20 bacilli were delivered to each animal using a Madison chamber aerosol generation device^3^.

### Oral glucose and insulin tolerance testing

Glucose and insulin tolerance were measured as previously described^3^. In brief, following an 8–12 h fasting period, glucose tolerance was measured by oral challenge with a 2 g/kg bolus of d-glucose (0.5 g/ml). Serum glucose concentrations were measured using the Freestyle Lite glucometer (Abbot) from a skin-prick site adjacent to the most peripheral vein on the ear pinna at 0, 30, 60, 90, and 120 min following administration. Similarly, insulin sensitivity was measured following the SC injection of 0.5 units/kg of regular-acting human recombinant insulin (Humulin-R, Eli Lilly). Glucose concentrations were measured at 0, 25, 50, 75, and 100 min following insulin injection.

### Metabolic phenotyping

Peripheral blood mononuclear cells were isolated from whole blood by density gradient centrifugation with lympholyte mammal (1.086 gm/cm^3^) according to manufacturer instructions (Cedarlane). Single-cell suspensions from BAL were prepared by centrifugation at 500×*g* followed by RBC lysis with Gey’s solution. 2 × 10^5^ or 4 × 10^5^ PBMCs and 1 × 10^5^ BAL cells suspended in Seahorse XF assay media (Agilent Technologies, Inc), were seeded onto CellTak (Corning Inc.) coated 8 or 24 well cell culture microplates, respectively. Cell adherence to microplates was achieved prior to assaying by centrifuging plates at 500×*g* for 5 min with no brake followed by a 30-min incubation at 37 °C in the absence of CO_2_. OCR and ECAR were measured using a Seahorse XFp or Seahorse XFe24 bioanalyzer (Agilent Technologies, Inc). OCR and ECAR were measured basally and following the injection of 1 μM oligomycin, 2 μM fluorocarbonyl cyanide phenylhydrazone (FCCP), 0.5 μM rotenone + antimycin A, and 50 mM 2-deoxyglucose.

### Flow cytometry

Peripheral blood mononuclear cells and BAL single-cell suspensions were prepared for antibody surface staining by seeding 1 × 10^6^ cells in FACS buffer, PBS + 1% BSA + 0.1% sodium azide, into 96 well round-bottom plates. Fc blocking was performed in FACS buffer + guinea pig IgG + rabbit IgG on a rocker at 4 °C for 30 min prior to antibody staining. Fluorochrome-conjugated antibodies including CD4, CD8 (Bio-Rad Laboratories Inc.), and MHCII (hybridoma line producing mAb IVA12^32^) were prepared in FACS blocking buffer and added to cells for 20 min at 4 °C on a rocker. Live/dead exclusion was assessed by staining with either Live/Dead aqua or 7-AAD. Data was collected with an LSR II (BD Biosciences) flow cytometer maintained within BSL-3 facilities and the data was analyzed using FlowJo software (FlowJo, LLC).

### Mitochondrial staining

Mitochondrial membrane potential was evaluated by incubating PBMCs with 25 nM DiOC_6_(3) (ThermoFisher Scientific) for 30 min at 37 °C + 5% CO_2_. Mitochondrial mass was assessed by staining PBMCs with 100 nM mitotracker deep red (ThermoFisher Scientific) for 30 min at 37 °C + 5% CO_2_. To quantify mitochondrial superoxide production, MitoSOX red (ThermoFisher Scientific) was suspended at a final concentration of 2.5 μM in HBSS and incubated with PBMCs for 10 min at 37 °C with CO_2_. To obtain a positively stained MitoSOX control, PBMCs were incubated with 93 nM tert-butyl hydroperoxide for 30 min at 37 °C + CO_2_ prior to MitoSOX staining. All samples were analyzed using an LSR II (BDBiosciences) flow cytometer maintained within BSL-3 facilities and the data was analyzed using FlowJo software (FlowJo, LLC).

### GSH/GSSG ratio

GSH/GSSG ratios were analyzed in isolated PBMCs during acute, subacute, and chronic stages of Mtb infection by luciferin detection using the GSH/GSSG-Glo™ Assay kit (Promega). 2.5 × 10^5^ live PBMCs were assayed in a 96 well white-walled plate, according to manufacturer instructions, and luminescence was measured on a BioTek Synergy2 instrument.

### qRT-PCR

The right caudal lung lobe was collected post-mortem from metformin and untreated animals and minced in RNAlater (Qiagen), incubated at 4 °C for 24 h, then frozen at − 80 °C for extraction at a later date. Approximately 50 mg of minced tissue was homogenized with a bead beater in RLT buffer then digested with proteinase K for 30 min at 55 °C. Nucleic acid was extracted with an RNeasy mini kit (Qiagen). The nucleic acid was eluted, and DNA was digested in solution with DNase I (Qiagen) for 30 min at RT then re-purified on the RNeasy mini columns. The resulting RNA was quantified by nanodrop and integrity determined by agarose gel electrophoresis. 1 μg of RNA was reverse transcribed to cDNA and quantitative RT-PCR was performed with the SYBR Green detection kit (Bio-Rad) and signal detected in the Bio-Rad CFX-96 real-time thermal cycler. Each reaction was performed in duplicate for each animal with 0.2 mmol/L of each primer and 50 ng of cDNA template. The gene expression was normalized using two reference genes- hypoxanthine–guanine phosphoribosyltransferase (HPRT) and TATA-box binding protein (TBP), which were validated for consistent expression with these experimental conditions. The primer design and sequences for guinea pig TNFα, IL-6, IL-12p40, IL-1β MCP-1, TGFβ, IL-10, IFNγ, IL-17A, and FOXP3 are thoroughly described in our previous work^8^.

### Bacterial burden/CFU

Lung, spleen, and liver were weighed and diluted 1:10 in PBS prior to homogenization. Serial dilutions of tissue homogenates were performed in PBS and plated on BD Difco™ 7H11 agar (VWR). Following 3–6 weeks of incubation at 37 °C, colony-forming units were counted, and CFUs per gram of tissue was calculated.

### Histology

Tissue sections fixed with 4% paraformaldehyde were paraffin embedded, sectioned at 5 μm, and stained with hematoxylin and eosin. Total and lesion area of lung, spleen, and liver were quantified using Stereo Investigator area fraction fractionator software (MBF Bioscience) and the Nikon Eclipse 80i microscope. The area of inflammation relative to the area of normal tissue parenchyma was estimated from representative lung, liver, and spleen tissue sections evaluated at 200× magnification. A total of 20–25 fields were randomly selected by the computer, and a counting frame (2000 μm^2^) containing probe points with a grid spacing of 200 μm was used to define the areas of interest (lesions and lungs). Lesion burden was calculated as ratio of tissue area to lesion area and expressed as a percentage of lesion to tissue^3^.

### Data analysis

Lesion burden and metabolic phenotyping data set was normalized to the initial time point of mock treated. Tukey’s IQR method was used to identify outliers in data sets. Based on data sets, Two-way ANOVA or T-test were used to determine differences between groups. When necessary, data was log transformed to achieve normality and homoscedasticity of residuals or data. If normality or homoscedasticity could not be achieved, data was analyzed using non-parametric methods. Post-hoc comparisons methods were Tukey, Sidak's, or Mann–Whitney multiple comparisons test, respectively. Data was analyzed with Graph Pad Prism v.8.

### Ethics statement

All animal experiments were performed in accordance with the National Research Council's Guide for the Care and Use of Laboratory Animals and were approved by the Animal Care and Usage Committee at Colorado State University.

## Discussion

In these studies, we tested the hypothesis that reversing non-diabetic hyperglycemia in response to Mtb infection with metformin would improve clinical outcomes in Mtb infected guinea pigs. When metformin treatment was initiated 28 days prior to Mtb infection, treated guinea pigs had significantly reduced lung lesion and bacterial burdens in the chronic stages of infection compared to untreated animals. This improvement corresponded with improved systemic insulin sensitivity, similar to the expected treatment response in diabetic patients. However, significant improvements in fasting blood glucose levels or glucose tolerance following Mtb infection were not achieved following metformin treatment. These data suggest that although metformin improved TB disease outcome, maintenance of systemic glucose tolerance alone was not responsible for these effects and suggests an alternative mechanism of action.

Multiple reports have shown the potential use of metformin as an immune-modulatory drug in the treatment of various chronic inflammatory diseases^14,17,19,33^. These effects are, in part, due to its partial inhibition of complex I of the electron transport chain, which leads to downstream signaling events that regulate cell metabolism, growth, and function in T cells and other cell types^34−36^. Here, we show that following chronic Mtb infection, metabolic flux through mitochondrial respiration and glycolysis are dramatically upregulated in T cells isolated ex vivo from untreated Mtb infected guinea pigs. This response was not unexpected given the systemic inflammation and resulting acute and chronic immune cell activation associated with active TB disease^37,38^. Interestingly however, our results are in contrast with recent studies performed by Russell et al., in which they report a decrease in CD8^+^ T cell mitochondrial respiration following Mtb infection in mice^24^. It is therefore, important to note that among differences in animal models and evaluation of PBMCs versus isolated CD8^+^ T cells, we found that different stages of disease corresponded to fluctuating metabolic phenotypes, which may help to explain variability within the literature. Furthermore, we show that during chronic infection, PBMC metabolism is restored to normal in metformin but not untreated animals. This was not due to variations in the percentages of circulating immune cells, but rather resulted from inherent cellular metabolic changes in response to metformin treatment. These findings are consistent with those observed in mouse models of systemic lupus erythematosus, in which metformin combined with 2-deoxyglycose normalized PBMC metabolism and resulted in decreased immune activation and the subsequent resolution of clinical disease^19^. We therefore, reasoned that changes in peripheral blood T cell metabolism reflect the waxing and waning of inflammation in response to persistent antigen stimulation, which was normalized by metformin treatment^39−41^. These data are consistent with similar studies and suggest that the beneficial effects of metformin treatment were not due to the restoration of normal systemic glucose metabolism, but rather a direct immunomodulatory effect on immune cells responding to Mtb infection^24,42^.

In addition to similar work done in TB, non-TB studies also show that metformin treatment dampens inflammation and limits tissue destruction in a variety of communicable and non-communicable diseases by limiting the differentiation and pro-inflammatory potential of T lymphocytes^28,29^. Although we were limited in our ability to directly assess T cell function and differentiation of other T cell subsets due to the lack of available guinea pig reagents, we evaluated direct parameters of CD4^+^ and CD8^+^ T cell energy metabolism that correspond to their function, including mitochondrial membrane potential and mitochondrial superoxide production. Mitochondrial membrane potential, a measure of the gradient of hydrogen ions in the mitochondrial inner membrane space, is the primary driver of ATP production by the cellular electron transport chain. Inhibition of complex I has been shown to decrease mitochondrial membrane potential and lead to the downregulation of anabolic metabolism that is associated with effector T cell growth and function^31,43,44^. In addition, a recent study found that T cells with a high mitochondrial membrane potential correspond to highly differentiated effector cells with increased metabolism, while those with low membrane potential are less differentiated cells that possess the ability to self-renew and retain immunological plasticity^31^. In our studies, peripheral CD4^+^ and CD8^+^ T cells isolated from guinea pigs treated with metformin had significantly reduced membrane potential compared to untreated animals during acute and chronic stages of Mtb infection. These data suggest that metformin, by maintaining a lower mitochondrial membrane potential, has the potential to influence the differentiation and function of T cells responding to Mtb infection, which is currently being investigated in our laboratory.

Mitochondrial superoxide production is also critical for the functional response of T cells and is found to play dual roles in both the promotion of effector functions and/or oxidative damage that can ultimately lead to cell death^30,31,45^. A direct link between membrane potential and superoxide has been described in systemic lupus erythematosus (SLE), where high membrane potential and inadequate ATP production correspond to obstructed electron flow, resulting in electron leakage and ROS production^30^. Due to the increased respiratory rates observed in response to Mtb infection, we hypothesized that mitochondrial superoxide would also be increased in T cells from untreated Mtb infected guinea pigs, while animals that received metformin would display reduced superoxide. Surprisingly, metformin significantly enhanced mitochondrial superoxide production by CD4^+^ T cells during chronic infection. This result was unexpected given that both mitochondrial respiration and membrane potential were reduced in T cells from metformin treated animals. One possible explanation is that increased mitochondrial superoxide is associated with maintenance of T cell functionality during chronic infection rather than oxidative damage and dysfunction^46,47^.

To assert whether increased superoxide production was indicative of increased oxidative stress we measured the relative abundance of GSH: GSSG. Despite the increase in superoxide by CD4^+^ T cells from metformin treated animals, GSH: GSSG ratios remained consistent between both groups. These data provide further evidence that the influence of metformin on CD4^+^ superoxide production may function to maintain important cell signaling events in CD4^+^ T cells^48^.

The differential expression of cytokine genes in metformin treated and untreated guinea pigs by mRNA analysis also supports the hypothesis that metformin elicits its effects by altering immune cell function during Mtb infection and is consistent with findings by others^24^. We found that animals treated with metformin had dramatically decreased expression of pro-inflammatory cytokines. Moreover, although we observed increased expression of FOXP3, there was no difference in the production of regulatory cytokines, suggesting that the anti-inflammatory response was due to decreased pro-inflammatory cytokine production by effector cells. Taken together, these data provide strong evidence that metformin exerts its immunomodulatory effects directly on T cells during Mtb infection, resulting in a decreased pro-inflammatory response which explains the decreased lung inflammation and evidence of lesion resolution seen primarily in treated animals.

Importantly however, although metformin significantly reduced disease manifestation during chronic stages of infection in our studies, these findings did not correspond to increased guinea pig survival. In contrast to decreased lung lesion burden, there were no significant differences in the extrapulmonary lesion or bacterial burden in metformin treated animals, which likely explains the lack of improved survival. In addition, these studies did not assess the use of metformin as a therapy for pre-existing infection or as adjunctive therapy in combination with antimicrobial drug treatment of TB^49^. These are critical questions that will be evaluated in our future work and must be taken into consideration before metformin is evaluated as a practical host-directed therapy in humans.

Together, these studies show that alterations to host metabolism play an important role in the progression and severity of TB disease. Although we were unable to directly link the impact of non-diabetic hyperglycemia to TB disease pathogenesis using metformin, we discovered novel immune metabolic correlates of protection. Moreover, our results are consistent with previous reports showing metformin’s protective efficacy against Mtb infection in mice^14,18,24^. Our results and others, therefore, warrants further evaluation of metformin and other drugs that target immune cell metabolism in the ongoing discovery and development of novel TB therapeutics^50^.

## Supplementary information

Supplementary Figures.
