# Supplementary material for: Metformin enhances protection in guinea pigs chronically infected with Mycobacterium tuberculosis
Source: Sci Rep. 2020 Oct 1;10:16257. doi: 10.1038/s41598-020-73212-y (PMC7530990; doi:10.1038/s41598-020-73212-y)
Supplement: Supplementary file 1 — Supplementary Figures. [file 41598_2020_73212_MOESM1_ESM.docx]

**Supplemental Information**

***Metformin enhances protection in guinea pigs chronically infected with Mycobacterium tuberculosis***

Jessica D. Haugen Frenkel^1^, David F. Ackart^1^, Alexandra K. Todd^1^, James E. DiLisio^1^, Siana Hoffman^1^, Samantha Tanner^1^, Dilara Kiran^1^, Megan Murray^2^, Adam Chicco^3^, Andrés Obregón-Henao^1^, Brendan K. Podell^1^, Randall J. Basaraba^1^

Correspondence to: [basaraba@colostate.edu](mailto:basaraba@colostate.edu)


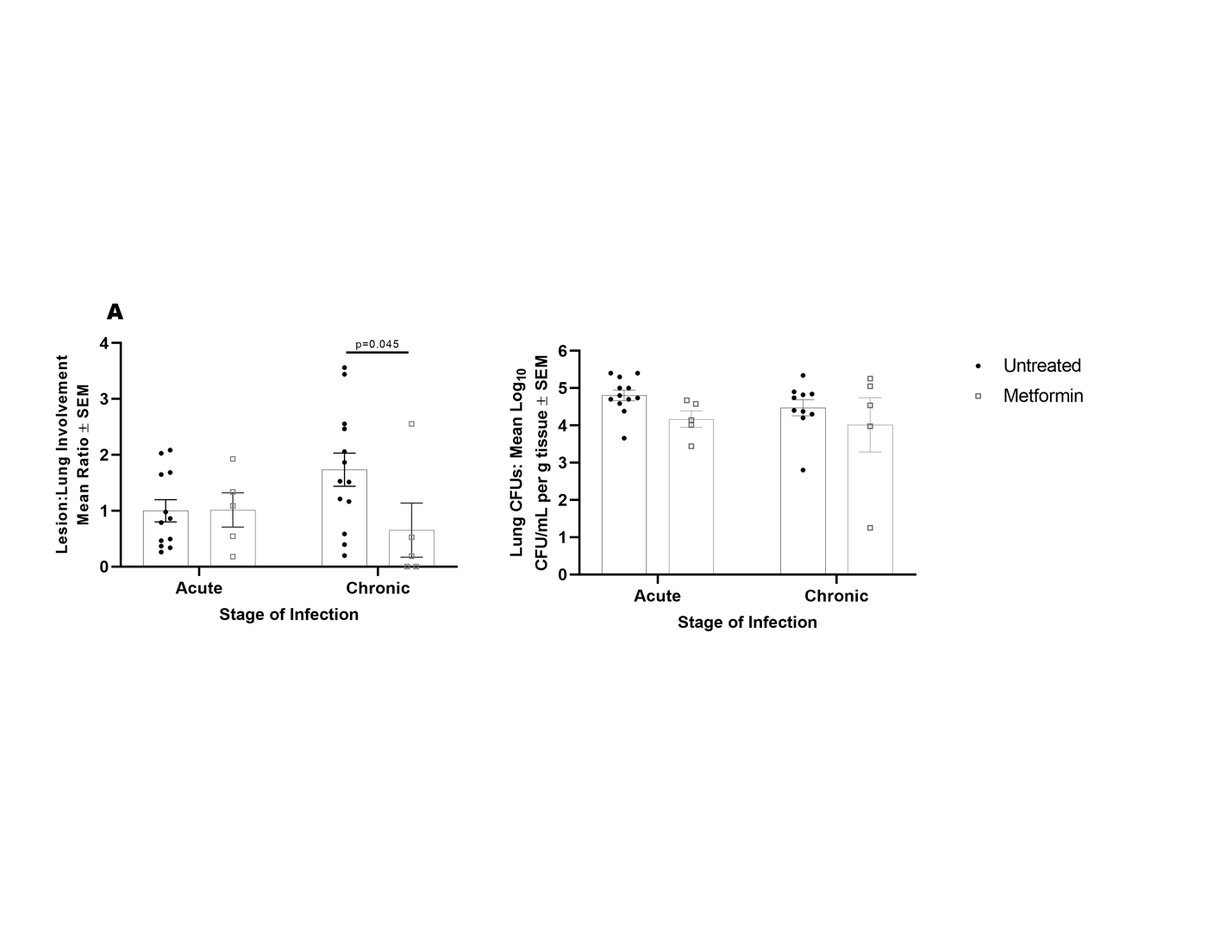


**Supplemental Figure 1.** **Metformin enhances host resistance when administered concurrent with Mtb.**During acute and chronic stages of infection lungs were harvested from euthanized, Mtb infected untreated and metformin-treated guinea pigs, n=14, to evaluate lesion burden (A) and bacterial burden (CFUs) (B). Stereo Investigator software was used to calculate the percent of lesion to uninvolved tissue in guinea pig lungs. Percent lesion involvement was normalized to acute untreated values. Colony forming units were generated by plating homogenized lung on 7H11 agar plates and were quantified per gram of tissue. (B) Two outliers were found using Tukey IQR method. All outliers were greater than 2.0 times the IQR. Mann-Whitney test was used to determine significances between treatments within stage of infection. * p ≤ 0.05.


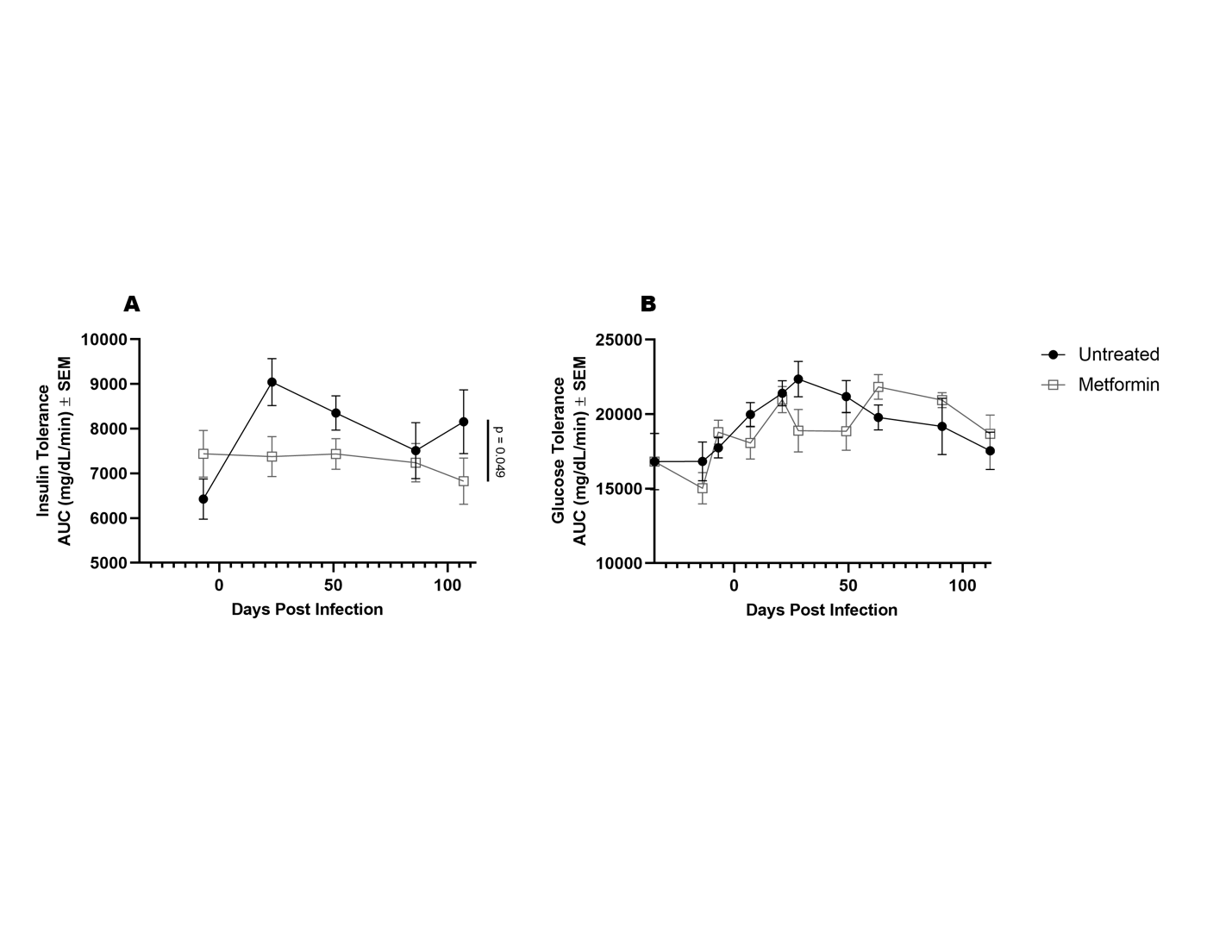


**Supplemental Figure 2. Oral glucose and insulin tolerance test.** On days -7, 23, 51, 86, and 107 following Mtb infection, guinea pigs were subjected to an insulin tolerance test by injecting 0.5 units/kg human recombinant insulin SC (A). Blood glucose concentrations were measured at 0, 25, 50, 75, and 100 minutes post insulin injection. On days -35, -14, -7, 7, 21, 28, 49, 63, 91, and 112 following infection guinea pigs were subjected to an oral glucose tolerance test by challenging fasted animals with a 2g/kg bolus of D-glucose (B). Similarly, blood glucose was measured at 0, 30, 60, 90, and 120 minutes post oral glucose challenge. The area under the curve (AUC) was calculated for each treatment group at each individual timepoint (A-B). Graphs represent the corresponding averages of the AUC of either untreated or metformin treated guinea pigs at each timepoint. Significance of treatment effect was found using a 2-factor ANOVA. There was no significant difference in glucose tolerance between mock- and metformin-treated Mtb infected pigs over the course of infection (B). n=12.


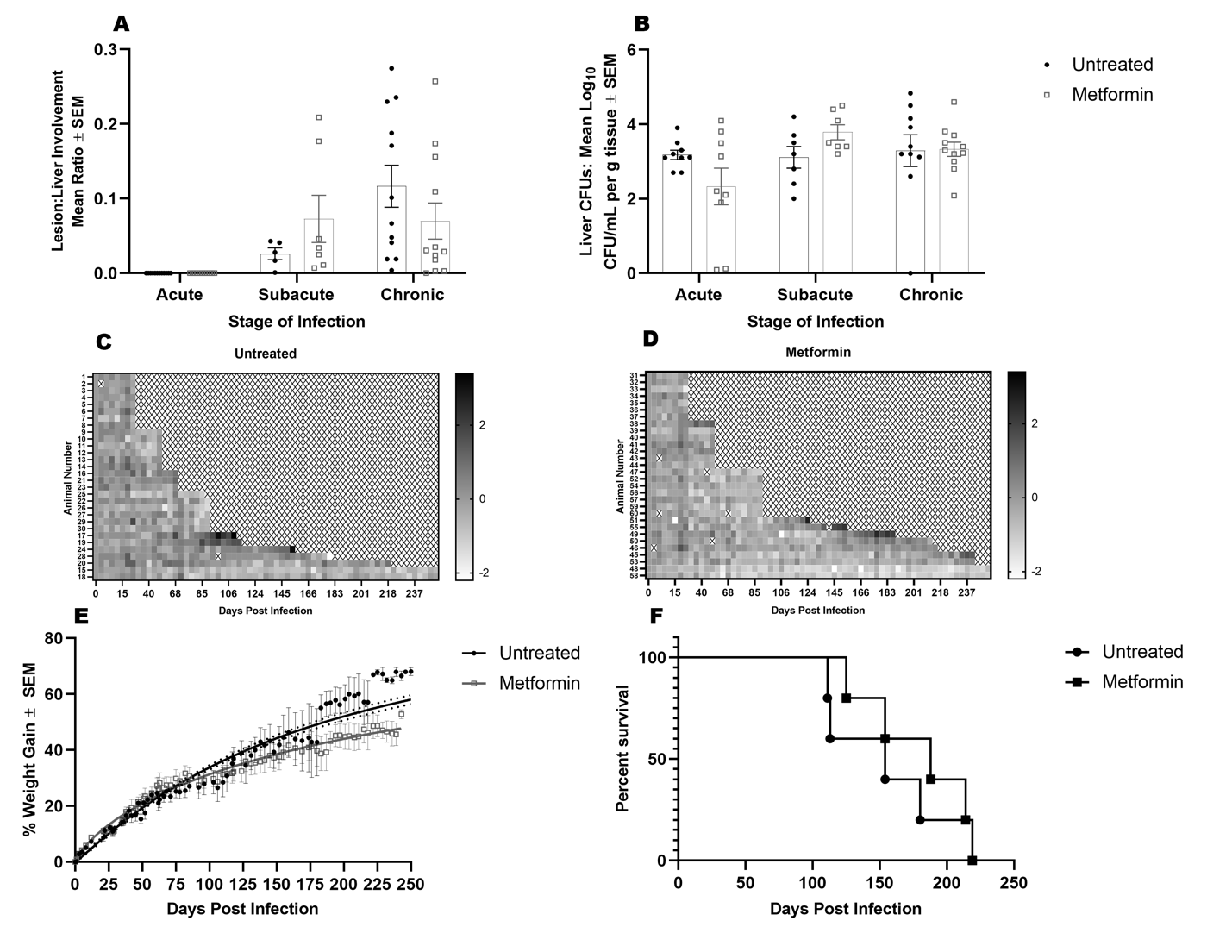


**Supplemental Figure 3. Extra-pulmonary lesion and bacterial burden and clinical parameters.** During acute, subacute, and chronic stages of infection livers were harvested from euthanized, Mtb infected untreated and metformin-treated guinea pigs to evaluate lesion (A) and bacterial burden (CFUs) (B). Stereo Investigator software was used to calculate the percent of lesion to uninvolved tissue in guinea pig livers (A). Percent lesion involvement was normalized to acute untreated. Colony forming units were generated by plating homogenized liver on 7H11 agar plates and were quantified per gram of tissue (B). Clinical signs of disease were assessed in untreated (C) and metformin-treated (D) Mtb infected guinea pigs by tracking and body temperature, body weight (E), and animal survival (F) up to 250 days following Mtb infection.


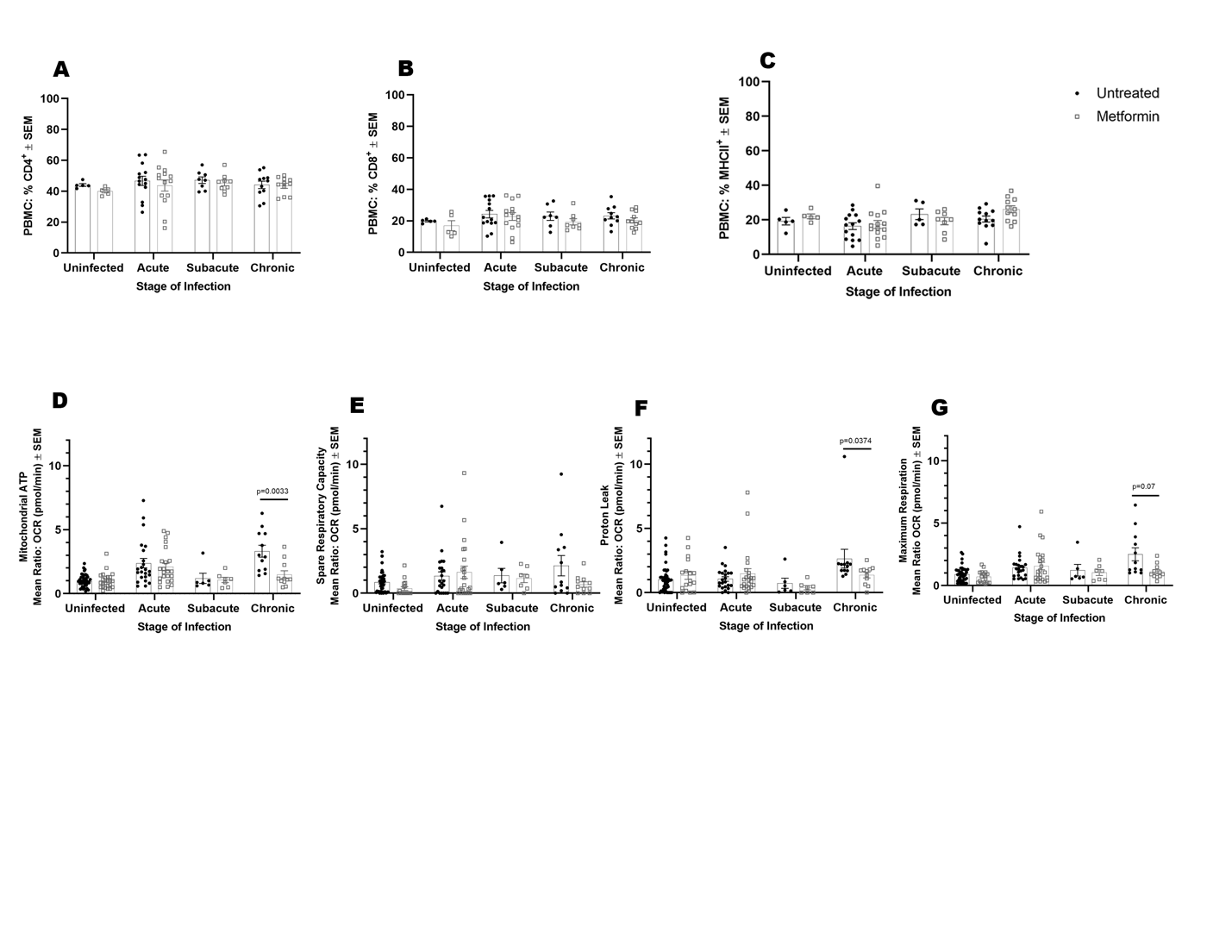


**Supplemental Figure 4. CD4+ and CD8+ T cell and MHCII+ cell analysis and metabolic profiles.** Flow cytometry analysis was used to quantify the percentages of CD4^+^ (A), CD8^+^ (B), and MHCII^+^ (C) cells from untreated and metformin-treated guinea pigs prior to and following low dose aerosol infection with Mtb at acute, subacute, and chronic stages of infection. Seahorse extracellular flux analysis and the use of metabolic inhibitors (oligomycin, rotenone, and antimycin A) and uncouplers (FCCP) were used to measure mitochondrial ATP production (D), spare respiratory capacity (E), proton leak (F), and maximal respiration (G). Data was normalized to the mean of the uninfected untreated group between two independent experiments. Mann-Whitney test was used to determine differences between groups and within each stage of infection. * p ≤ 0.05 ** p ≤ 0.01.


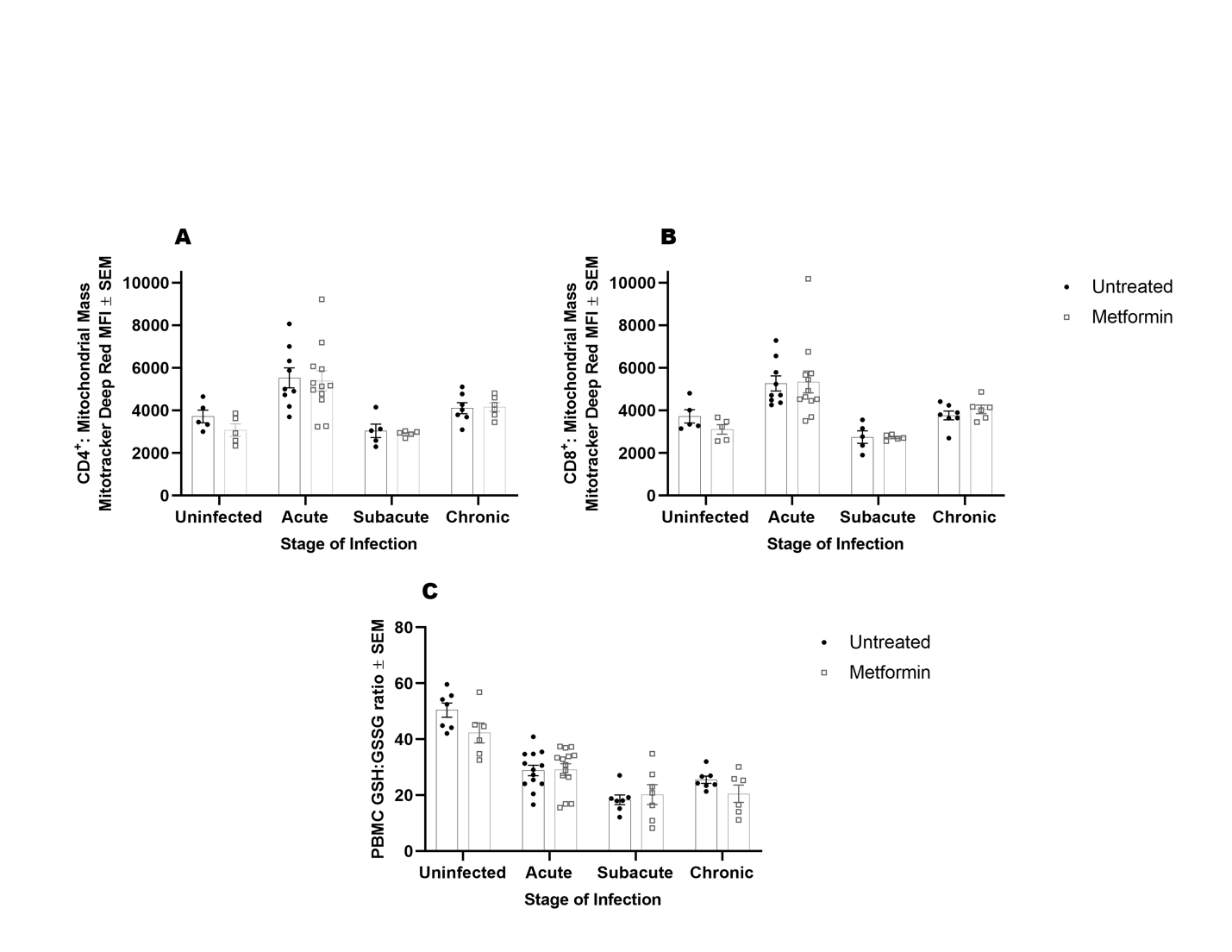


**Supplemental Figure 5. CD4+ and CD8+ T cell mitochondrial mass and oxidative stress status.** Peripheral blood mononuclear cells were isolated prior to and during acute, subacute, and chronic stages of Mtb infection from mock- and metformin-treated guinea pigs. Mitochondrial mass of PBMC derived CD4+ (A) and CD8+ (B) T cells was evaluated using mitotracker deep red staining (100nM) and quantified by median fluorescence intensity by flow cytometry analysis. GSH to GSSG ratios were measured in PBMCs by luciferin detection using a GSH/GSSG-Glo Assay kit (C). N=6-13 per group per timepoint.


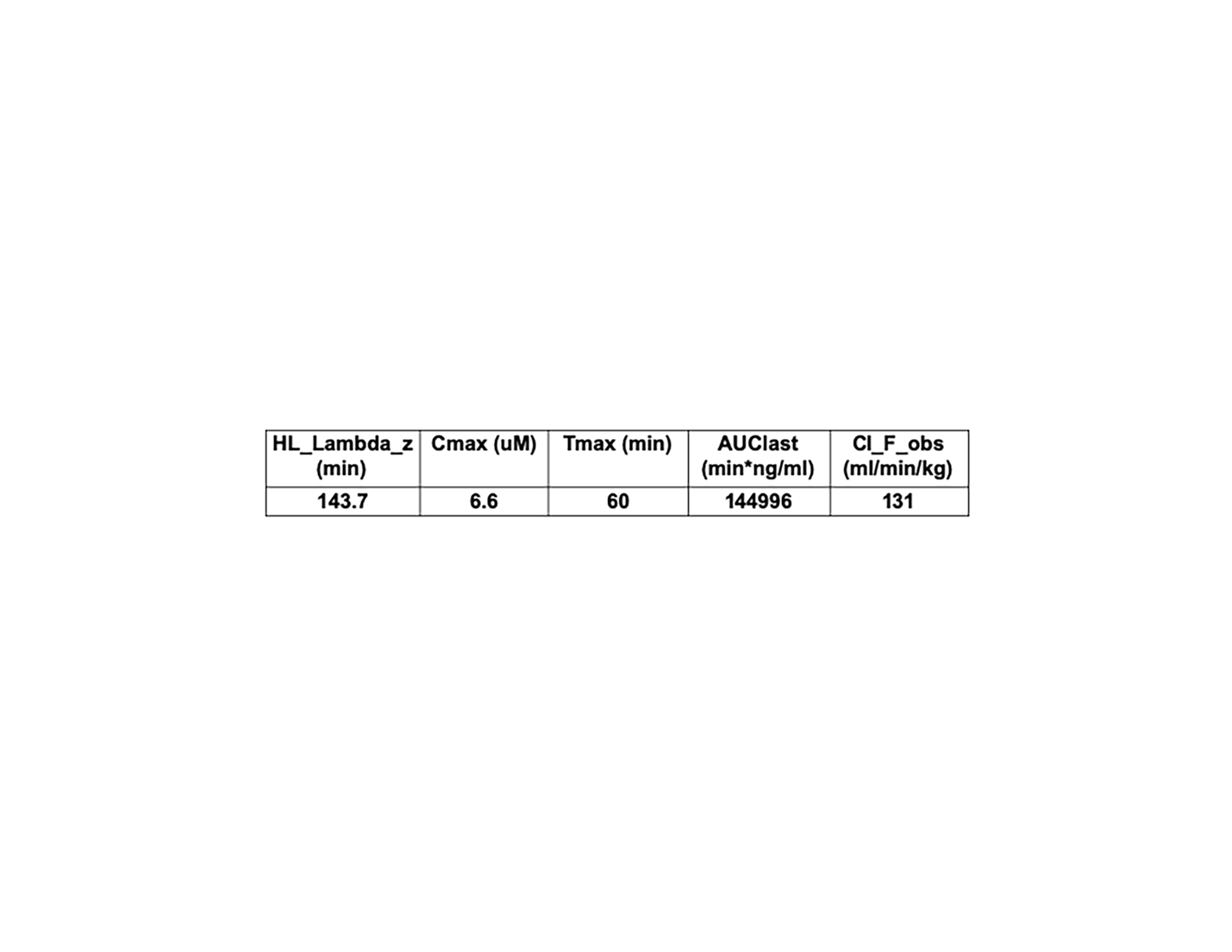


**Supplemental Figure 6.** **Metformin PK/PD.** Animals received oral dose of metformin as described in Methods Section. Whole blood samples were obtained from an indwelling venous catheter at 0, 5, 15, 30, 60, 120, 240, 360, and 480 minutes post metformin administration. Plasma was collected and metformin concentration determined by liquid chromatography-tandem mass spectrometry. Plasma concentration time-course measurements were analyzed by noncompartmental analysis.
